# Processing cataphors: Active antecedent search is persistent

**DOI:** 10.3758/s13421-021-01176-z

**Published:** 2021-05-11

**Authors:** Anna Giskes, Dave Kush

**Affiliations:** 1 Institute of Language and Literature, NTNU–Norwegian University of Science and Technology, Edvard Bulls vei 1, 7491 Trondheim, Norway; 2 Department of Language Studies, University of Toronto, Scarborough, Canada

**Keywords:** Coreference, Cataphora, Syntactic processing, Prediction, Active parsing, Self-paced reading

## Abstract

Cataphors precede their antecedents, so they cannot be fully interpreted until those antecedents are encountered. Some researchers propose that cataphors trigger an active search during incremental processing in which the parser predictively posits potential antecedents in upcoming syntactic positions (Kazanina et al., *Journal of Memory and Language, 56*[3], 384–409, 2007). One characteristic of active search is that it is persistent: If a prediction is disconfirmed in an earlier position, the parser should iteratively search later positions until the predicted element is found. Previous research has assumed, but not established, that antecedent search is persistent. In four experiments in English and Norwegian, we test this hypothesis. Two sentence completion experiments show a strong off-line preference for coreference between a fronted cataphor and the first available argument position (the main subject). When the main subject cannot be the antecedent, participants posit the antecedent in the next closest position: object position. Two self-paced reading studies demonstrate that comprehenders actively expect the antecedent of a fronted cataphor to appear in the main clause subject position, and then successively in object position if the subject does not match the cataphor in gender. Our results therefore support the claim that antecedent search is active and persistent.

## Introduction

Incremental sentence processing makes use of active or predictive parsing strategies in multiple linguistic domains and at different granularities (Kuperberg & Jaeger, 2016). Comprehenders appear to use a wide range of contextual information to make syntactic and lexical-semantic predictions during processing. A central question in understanding prediction in specific constructions is whether processing is governed by construction-specific subroutines or by more general active parsing mechanisms. In this study, we test whether a property that is often assumed for filler-gap processing, the showcase for active dependency completion, is present in the active search for an antecedent triggered by cataphors.

Cataphors, such as *she* in (1), precede their antecedent. As a result, the parser cannot immediately interpret a cataphor as soon as it is seen: it must be held ‘unresolved’ in memory until the antecedent is found later in the string and a referential dependency between cataphor and antecedent can be established:

1. Before *she*_i_ left the library, Mary_i_ thanked John for his help.

There is evidence that upon encountering a cataphor in a preposed subordinate clause, the parser predictively posits coreference between the cataphor and the upcoming main clause subject. For example, van Gompel and Liversedge (2003) presented readers with sentences such as (2), in which the cataphor (*he/she*) was manipulated to match or mismatch the gender of the main clause subject (*the boy*). Readers slowed down when the gender of the subject did not match the gender of the cataphor, as in (2b), as compared with (2a); van Gompel and Liversedge interpreted this *gender-mismatch effect* as reflecting violation of posited coreference:

(2) a. When *he* was at the party, the boy cruelly teased the girl during the party games.
  b. When *she* was at the party, the boy cruelly teased the girl during the party games.

Recently, the expectation of coreference with the upcoming subject has been characterized as reflecting a more general *active* search strategy that the parser adopts when processing prospective dependencies (Ackerman, 2015; Drummer & Felser, 2018; Kazanina et al., 2007; Pablos et al., 2015; Patterson & Felser, 2019) such that it predictively posits expected constituents in the closest possible syntactic positions as the sentence unfolds. This characterization of cataphor resolution as an active search is based on analogy to filler-gap dependencies, such as *wh*-dependencies.

In filler-gap dependencies, displaced elements (*fillers*) must be integrated at later *gap* positions. For example, the filler *who* in (3) is ultimately interpreted as the complement of the preposition *to*:

(3) My brother wanted to know *who* Ruth will bring us home to __ at Christmas.

When the incremental parser first encounters *who,* however, it does not know the true location of its corresponding gap. Research shows that the parser responds to this uncertainty by actively positing the gap in the closest syntactic position consistent with the incremental input (active gap-filling; Frazier, 1987). For example, Stowe (1986) found that readers were surprised by direct object *us* in (3), where readers were searching for a gap in the embedded clause, compared with sentences like (4), where no gap exists:

(4) My brother wanted to know *if* Ruth will bring us home to Mom at Christmas.

Stowe reasoned that this *filled-gap effect* was expected if the parser first predicts the gap for *who* as the direct object of *bring* in (3). Additional evidence for active gap filling is ample (e.g., Frazier & Clifton Jr., 1989; Frazier & d’Arcais, 1989; Garnsey et al., 1989; Kaan et al., 2000; Lee, 2004; Nicol & Swinney, 1989; Omaki et al., 2015; Traxler & Pickering, 1996).

If active gap-filling is driven by a pressure to discharge *wh*-dependencies as quickly as possible, one would expect the strategy to be *persistent:* If the parser’s initial prediction is foiled, it should iteratively posit a gap in the next closest syntactically available position until the real gap is encountered (Clifton & Frazier, 1989). Active gap-filling is implicitly understood as persistent, though persistence has not been demonstrated within a single study. Nevertheless, the variety of syntactic positions in which previous research has found evidence for active gap-filling is consistent with persistence. The vast majority of research observed effects of active gap filling in direct object position, which implies a search that has continued after having encountered a filled subject gap. Filled-gap effects have also been observed in subject position (Lee, 2004; Wagers & Pendleton, 2016), and (suggestively) past the direct object position (Boland, Tanenhaus, Garnsey, & Carlson, 1995; Wagers & Phillips, 2014).

Cataphoric dependencies superficially resemble filler-gap dependencies in two important ways: First, the interpretation of the first element depends on properties of the second element. Second, the syntactic position of the second element cannot be predicted with certainty. These similarities have led researchers to suggest that antecedent search in cataphoric processing is driven by the same active parsing mechanism that underlies active gap-filling (Kazanina et al., 2007).

The similarities are provocative, but the extent to which the analogy is apt needs to be tested by evaluating whether various characteristics are shared for both dependency types. In the present study, we test whether active antecedent search behaves like the active search procedures underlying filler-gap processing in terms of persistence.

It is not clear that active antecedent search is persistent. Whereas filled-gap effects have been observed in different syntactic positions, studies of cataphor processing have almost exclusively looked for evidence of active antecedent search in subject positions. As such, the evidence is consistent with two hypotheses regarding persistence. Active antecedent search might behave like active gap-filling and persist past foiled predictions, positing a position for the potential antecedent throughout the unfolding sentence until a suitable antecedent is found. We term this the “persistent search” hypothesis. The persistent search hypothesis is attractive, but it is conceivable that it is incorrect, given idiosyncratic properties of cataphoric processing. One reason why the parser might *not* adopt a persistent strategy for cataphor-antecedent search is that an intrasentential antecedent is not syntactically obligatory, unlike gaps in filler-gap dependencies. If a sentence contains a filler that cannot be linked to a gap, the sentence is ungrammatical. In contrast, if a sentence contains a pronoun that lacks a discourse antecedent, the sentence might be pragmatically infelicitous, but not syntactically ill-formed. Moreover, whereas the chance of a correct prediction of a gap location increases with every prediction that is foiled, the chance that a suitable antecedent for a cataphor occurs stays equal at best as the sentence progresses. It is possible, if pronouns must be linked to prominent positions (Gordon et al., 1993), that the probability of finding an antecedent decreases the deeper into a sentence the parser goes, given the fact that prominent positions tend to occur early in sentences (e.g., Gordon & Hendrick, 1998). If the parser weighs the benefits of efficient, predictive dependency formation against the increasing risk of having to perform a costly reanalysis for each foiled prediction, persistent antecedent search throughout the sentence might not be worth the effort.

An alternative account is that a cataphor’s search for an antecedent reflects a specific single prediction that the antecedent will occur in a subsequent subject position. Under this limited prediction hypothesis, the prediction would not be driven by a persistent commitment by the parser to discharge the dependency as soon as possible, but rather by independent reasons to expect the antecedent in subject position.

There may be information-structural motivations for expecting the antecedent in a subject position. Since topics have a high likelihood of being pronominalized (Ariel, 1990; Gordon & Hendrick, 1998; Grosz et al., 1995), comprehenders may incrementally interpret the cataphor as topic of the sentence. Such an interpretation may motivate a prediction of the antecedent in a canonical topic position, which corresponds strongly with subjecthood (Gordon & Hendrick, 1998; Grosz et al., 1995; Gundel,1988; Vallduvı & Engdahl, 1996). Additionally, it has been suggested that comprehenders have a general preference for assigning pronouns to referents in subject position (Stevenson et al., 1994), even if factors such as discourse coherence, implicit causality, and voice are controlled for (Kehler & Rohde, 2013a, 2013b). These preferences in pronoun interpretation seem also to be reflected in frequency data from English corpora (Crawley et al., 1990; Hobbs, 1978). If the parser based its predictions merely on the statistical probability that pronouns are linked to subjects, it might preferentially predict the antecedent for a cataphor in subject position.

Much recent research on cataphor antecedent search has investigated the nature of the active search, often in explicit comparison with active gap-filling. Multiple studies (Aoshima et al., 2009; Drummer & Felser, 2018; Kazanina et al., 2007; Kazanina & Phillips, 2010; Pablos et al., 2015; Patterson & Felser, 2019; Yoshida et al., 2014) have focused on whether antecedent search exhibits sensitivity to binding constraints, such as Principle C (Chomsky, 1981). Some of these studies provide evidence that is consistent with the persistent search hypothesis. For example, Kazanina et al. (2007) compared processing at the subject of an adjunct-internal subject phrase *the talented young quarterback* in (5), when it matched or mismatched a possessive cataphor (*His/Her*):

(5) a. *His* managers chatted amiably with some fans while the talented, young quarterback…
  b. *Her* managers chatted amiably with some fans while the talented, young quarterback…

The researchers observed gender mismatch effects at the noun *quarterback*, suggesting that antecedent search looks for an antecedent in subject position of the adjunct clause. Similar effects have been found by Felser and colleagues in German (Drummer & Felser, 2018; Patterson & Felser, 2019).

The results of these studies are, in principle, compatible with both the persistent search hypothesis and the limited prediction hypothesis. According to the persistent search hypothesis, the adjunct-internal position occupied by *quarterback* is not the first position that the parser would have posited an antecedent in (5): the position occupied by *some fans* could have hosted the cataphor’s antecedent. The gender-mismatch effects at the adjunct-internal subject reflect persistent search if the parser first predicted an antecedent in the object position, and then revised this prediction later. Under the limited prediction hypothesis, the gender-mismatch effect would reflect that the parser’s first and only prediction for the antecedent site was in subject position of a subsequent clause. Such a single prediction for coreference with an (adjunct-internal) subject could be made for various reasons including abovementioned general subject preference, coherence, or even probabilities of coreference patterns in participants’ input. Given that both hypotheses can explain the results, previous experiments do not establish that readers in fact predict the antecedent in possible positions earlier than those in which the gender-mismatch effect was observed.

In order to test the Persistent Search Hypothesis, we tested whether we can find evidence for successive prediction of the antecedent within the same sentence type. We investigated the processing of cataphoric pronouns in two languages: Norwegian (Experiment 1) and English (Experiment 2). Our experiments asked whether we could find evidence for active search in both subject position and object position when the main subject does not provide a matching antecedent for a cataphor.

## Experiment 1 (Norwegian)

### Experiment 1A: Self-paced reading

#### Materials

We constructed 24 item sets (see Table 1) with a cataphor (*han/hun*, ‘he/she’) in a preposed subordinate clause. Using a 2 × 2 design, we manipulated gender match between the cataphor and a proper name (*Bjørn*) in the main clause, and the syntactic position of the proper name. In the subject conditions, the proper name occupied the main clause subject position; in the object conditions, the proper name occupied the argument position immediately following the main verb. Depending on the verb, this was the direct object (20 items) or indirect object (4 items). The gender of the matching cataphor was counterbalanced across items.

**Table 1:**
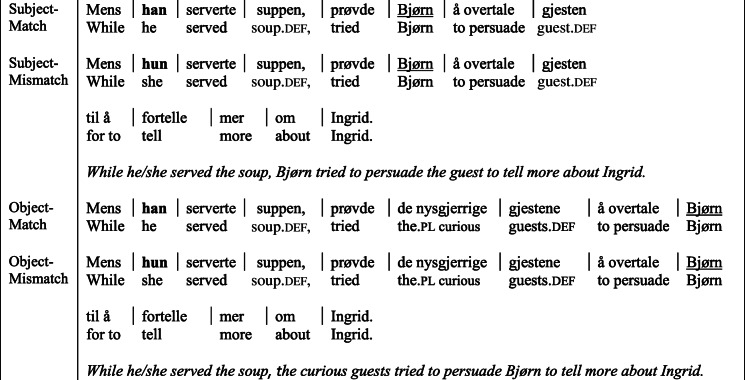
Experiment 1A: Example item set

*Note.* Regions separated by vertical bars.

The subordinated verbs were either intransitive, or transitive with an inanimate complement, so that no explicit additional referents were introduced in the subordinated clause. All referents introduced in the main clause were plausible as the subject of the subordinated predicates.

In the object conditions, the main clause subject was a plural Determiner Phrase (DP), thereby mismatching the cataphor in number. The subject DP, composed of a determiner, adjective, and a lexical noun, was divided into two regions: *Det+Adjective* and *Head Noun*, both of which carried plural-marking morphology. Dividing the subject in two regions increased the time between the first cue providing evidence of the subject number mismatch and the critical object region, making sure that the subject-number mismatch was well-established by the time participants reached the critical region.

Norwegian is a verb-second (V2) language (Den Besten & Edmondson, 1983). As a result, the preposed clauses were followed immediately by an auxiliary or subject control verb^1^ (as opposed to the subject in English studies). The main clause subject followed immediately after the auxiliary verb. Verbs in Norwegian do not agree with the main subject in either number or gender, so the auxiliary verb did not provide any morphological information that could have affected predictive processing of the subject. By using auxiliary verbs, we avoided providing information about the number of argument slots in the main clause. The processing of the main clause subject was thus not affected by item-specific plausibility of a cataphor interpretation that might follow from the main verb.^2^ Twenty of the items contained a third referent further on in the sentence that matched the gender of the cataphor in the mismatch condition (*Ingrid* in the sentences in Table 1), so that most cataphors were in principle resolvable.^3^

The item sets were distributed into four lists using a Latin square design and combined with 74 filler sentences of comparable length and complexity. The order was pseudo-randomized for each subject. Each sentence was followed by a yes/no comprehension question that never targeted the interpretation of the cataphor.

All materials were presented in *Bokmål*, the preferred written standard for 85%–90% of the Norwegian population and which all Norwegian students receive instruction in (Staalesen, 2014).

#### Procedure

The experiment was carried out on a MacBook Pro laptop using the Linger software package (Doug Rohde, MIT). Trials were presented using a self-paced reading moving window paradigm. Each trial started with a fixation cross, after which the sentence appeared, fully masked by dashes. Punctuation was masked as part of the preceding words, but spaces between words were visible. Participants made the words in the sentence appear one by one by pressing the space bar, and upon appearance of a word, the previous word was re-masked. Determiners and prepositions sometimes appeared together with the preceding or following word.

Each trial was followed by a comprehension question. Participants received direct feedback on the screen when they answered a question incorrectly. Halfway the experiment, a ‘break’ screen appeared. In addition to this built-in break, participants were instructed to take as many breaks as they wanted between trials.

Before the experiment, participants filled out a language background questionnaire and consent form. They received both oral and written instructions to read the sentences attentively and at a normal pace. After the instructions, participants completed a few practice items. The experiment took approximately 25 minutes, including instructions and breaks.

#### Participants

Sixty-seven native speakers of Norwegian (mean age: 27 years) with Bokmål as their preferred written form were recruited at NTNU Trondheim. They reported normal or corrected-to-normal eyesight, and no history of reading or language problems. They received a gift card worth NOK 100 for their participation.

#### Predictions

In line with previous research, we predict a gender-mismatch effect for the proper name in the subject conditions (i.e., longer reading times when the name mismatches the gender of the cataphor). Since effects in self-paced reading often occur in the region following the critical region, we expect the effect to occur in the critical name region or in the subsequent region.

For the object conditions, the persistent search hypothesis predicts a gender-mismatch effect at the critical name in object position or in the subsequent region. On the other hand, the limited search hypothesis predicts no gender-mismatch effect in the object conditions.

#### Analysis

Only data from participants who answered at least 80% of the comprehension questions correctly were included in the analysis. Five participants were excluded through this criterion. The remaining 62 participants had a mean accuracy of 87% (range: 81%–98%). Reading times under 150 ms and over 3,000 ms were excluded (<0.1% of the data).

We analyzed log-transformed reading times of the critical name regions and the spillover region using linear mixed-effects models (Baayen et al., 2008) implemented in the lme4 and lmerTest packages in R (R Core Team, 2015). We fitted separate models for the subject and object conditions, with match as a fixed effect. For all models, at least a random intercept and slope for subject, and a random intercept for item were included. Whenever models converged, we used maximal random effect structures (Barr et al., 2013). We analyzed subject and object conditions separately because the spillover regions across these conditions were not comparable. For the subject conditions, the spillover region was the main verb. For the object conditions the spillover region was in most cases a preposition. Running a single model would introduce potential confounds of length and content that were collinear with the subject and object manipulations.

#### Results

##### Question responses

The mean accuracy for the experimental items was 85.0%. This was slightly lower than the mean accuracy for the fillers (92.0%), but the difference was not significant (generalized mixed-effects model with participant and item as random effects: *z* = 1.49). A generalized mixed-effects model for the experimental items showed that there was no significant difference in accuracy between conditions either.

##### Reading times

**Fig. 1:**
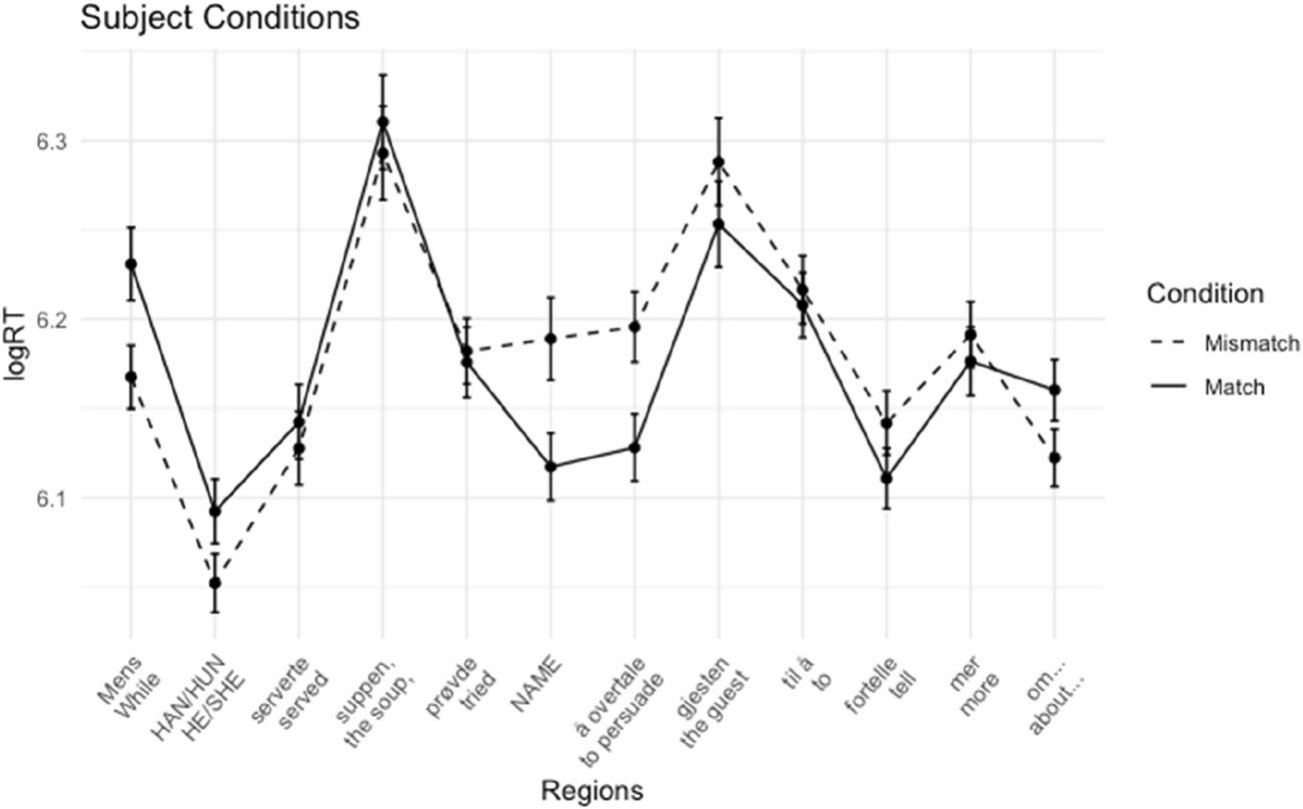
Experiment 1A: Average reading times + standard errors per region for subject conditions

**Fig. 2:**
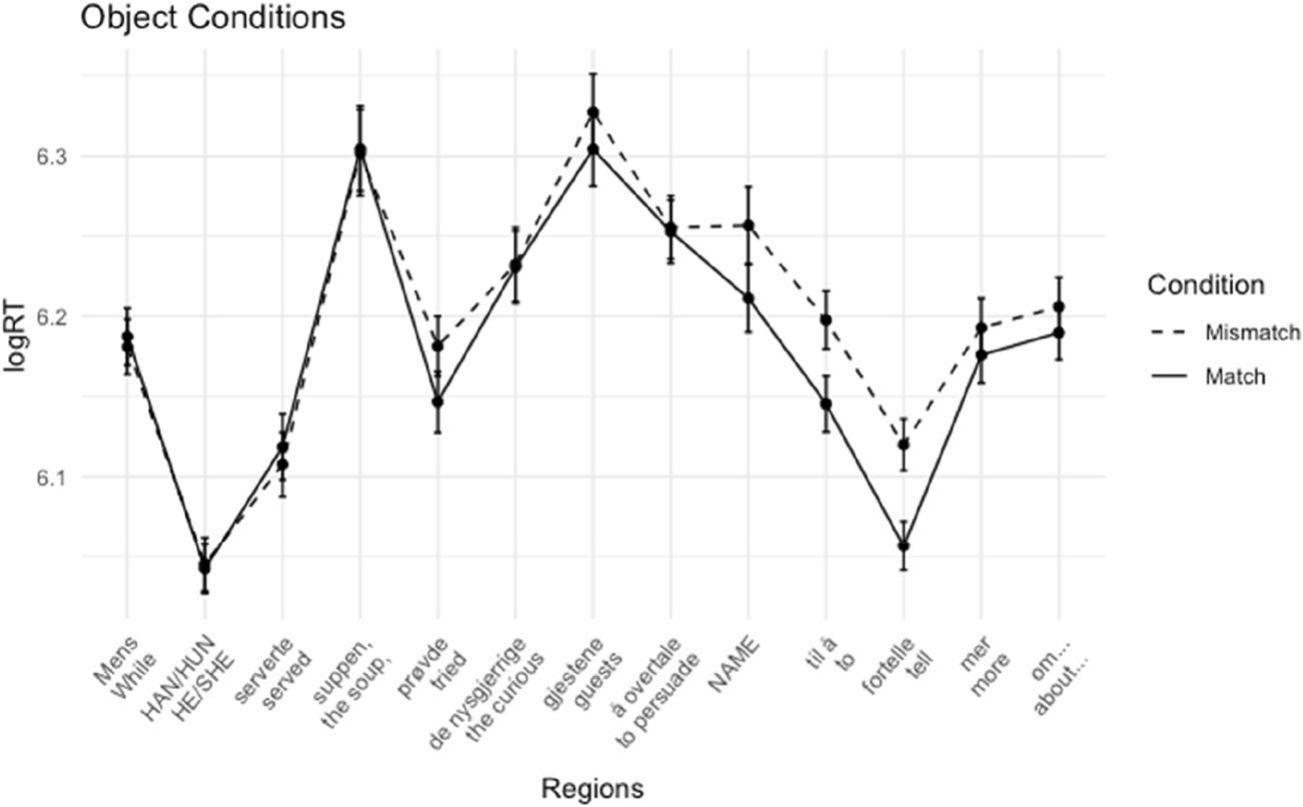
Experiment 1A: Average log reading times + standard errors per region for object conditions

Word-by-word log-transformed mean reading times per condition are plotted in Figs. 1 and 2. Mean reading times of the regions of interest are presented in Table 2.

**Table 2:** Experiment 1A: Mean reading times in ms (*SE*) for the critical name regions and the subsequent regions

	Name region	Spillover region
Subject-Match	488 (12)	494 (12)
Subject-Mismatch	546 (17)	532 (14)
Object-Match	548 (16)	497 (11)
Object-Mismatch	592 (20)	527 (13)

In the subject conditions, we observed longer reading times for the mismatch condition in the critical name region and the subsequent region. This gender mismatch effect was significant in both regions (name region: *t* = 2.75, *p* = .011; spillover region: *t* = 3.02, *p* = .004). For the object conditions, we also observed a slowdown for the mismatch cases in both the name region and the spillover region. However, in the name region, this trend did not reach significance (*t* = 1.50, *p* = .138). In the spillover region, the effect was significant (*t* = 2.15, *p* = .038). Although we did not analyze regions past the first spillover region, the effect seems to persist in the following region.

#### Discussion

The results from Experiment 1 show a replication of the well-established gender-mismatch effect in subject position: When the name in subject position mismatched the gender of the cataphor, readers slowed down at the name region, suggesting that they anticipated coreference with the cataphor. In addition, the results show a gender mismatch effect in the object conditions. In line with the persistent search hypothesis, these results suggest that when there was no matching antecedent in subject position, participants also actively posited coreference with the object position.

An alternative explanation for gender-mismatch effects is that the difficulty observed at the subject in mismatch condition reflects the introduction of a new referent. The rationale is that encountering a mismatching proper name will require introducing a new referent into the discourse representation of the sentence. In previous research, this potential confound has been addressed with additional control conditions, in which the cataphor was replaced by a first person singular pronoun (van Gompel & Liversedge, 2003) or a proper name (Kazanina et al., 2007). In these control conditions, the NP in main subject position had to be a new referent, so an index of the cost of referent accommodation was obtained by comparing the severity of the slowdown in these control conditions to the cataphor conditions. The results of these experiments show that the slower reading times in their cataphor-mismatch conditions cannot solely be explained as a slowdown caused by the introduction of a new referent. Since our experimental items are highly similar in structure to these studies, we consider it unlikely that referent accommodation underlies the mismatch effects we observed.

At this point, it appears that the effect in subject and object position may differ in size and timing. We observed a numerically smaller and possibly later effect in object position, only statistically significant in the spillover region. If this difference is meaningful, it could be due to a number of factors. Since the object effect occurs further downstream in the sentence, relatively more other incremental processes are being carried out when reaching the name region, most importantly the (syntactic and semantic) integration of the main clause verb and its argument structure. The parser may postpone or ‘spread out’ the reanalysis that is required after the mismatch, while also attending to the other ongoing processes (see Keshev & Meltzer-Asscher, 2020, for a similar account of ‘delayed reanalysis’).

Another possible account is that the difference in effect size reflects a different degree of commitment to the prediction. It is possible that the parser predicts subject coreference with more confidence, leading to a larger commitment to the prediction, and a larger disruption when it is foiled. This confidence could be motivated by any of the factors discussed in the Introduction that make subject coreference likely.

A third explanation for the smaller object effect could be that idiosyncratic features of individual test sentences may have biased readers against actively positing coreference with the object argument (e.g., due to semantic plausibility or subcategorization frequencies). Before reading the object position, participants have read the preposed clause, the subject, and the main verb, all of which allows readers to make a more informed assessment of the plausibility of coreference between the name and the cataphor in the object conditions than in the subject conditions. The potential influence of this item-specific plausibility is addressed in a sentence completion experiment, in which participants produced completions of the items, cut off before the name region.

### Experiment 1B: Sentence completion task

The aim of this experiment was to estimate item-specific probability of coreference between the cataphor and upcoming argument. Sentence completion tasks provide a less immediate response than self-paced reading responses, but they nevertheless give us an indication of comprehenders’ expectation of upcoming semantic and syntactic structure. If this expectation is driven by a persistent attempt to discharge the referential dependency created by the cataphor as soon as possible, speakers should prefer to introduce the antecedent immediately.

#### Materials

Twenty-four sets of preambles were created by truncating the item sets from Experiment 1A before the proper name. This resulted in four conditions (cataphor gender × cutoff point, see Table 3). The sets were distributed over four lists using a Latin square-design, and they were mixed with twenty-four items from an unrelated experiment, containing preambles with relative clauses.

**Table 3:** Experiment 1B: Example item set

Subject- masculine	Mens	**han**	serverte	suppen,	prøvde _____			
	While	he	served	the_soup,	tried			
Subject- feminine	Mens	**hun**	serverte	suppen,	prøvde _____			
	While	she	served	the_soup,	tried			
Object- Masculine	Mens	**han**	serverte	suppen,	prøvde	de nysgjerrige	gjestene	å overtale _____
	While	he	served	the_soup,	tried	the.PL curious	guests.DEF	to persuade
Object- feminine	Mens	**hun**	serverte	suppen,	prøvde	de nysgjerrige	gjestene	å overtale _____
	While	she	served	the_soup,	tried	the.PL curious	guests.DEF	to persuade

#### Procedure

The experiment was administered using Ibex Farm (Drummond, 2013). Participants were presented with the preambles one by one and were instructed to type in a text field below the preamble how they thought the sentence might continue. Participants were told that they could write whatever they choose, so long as the sentences were grammatically well formed.

#### Participants

Thirty-four native speakers of Norwegian (mean age: 35) that did not participate in the self-paced reading experiment volunteered for the experiment and participated via an online link. Two participants were excluded because of a large number of ungrammatical and nonsensical responses.

#### Coding and exclusion criteria

Responses that resulted in an ungrammatical sentence were excluded from the analysis (seven responses, <1% of the data). Furthermore, all responses (either to test or filler items) that were absurd were flagged to help us assess how seriously individual participants took the task. Almost all flagged responses belonged to two participants, who were excluded before analysis. The remaining participants gave the impression of having taken the task seriously: Their overall responses seemed not too far-fetched and were not written in an unusual register.

The responses were categorized based on whether and where a potentially co-referent element occurred. An element was considered potentially co-referent if (i) it was an animate definite NP matching the number and biological gender of the cataphor or a pronoun matching the gender, number, and person of the cataphor, and (ii) there was no compelling reason to assume that the intended referent was *not* the cataphor.^4^

Responses were classified as *immediate match* if the first available argument position (i.e., main clause subject position in subject conditions, and (in)direct object position on object conditions) was a matching potentially co-referent element; as *other match* if such an element occurred at another position; and *no match* if the response did not contain any such element. Possessive pronouns (e.g., *After he paid,* *his* *dog…*) were counted as *other match*.

#### Results

**Fig. 3:**
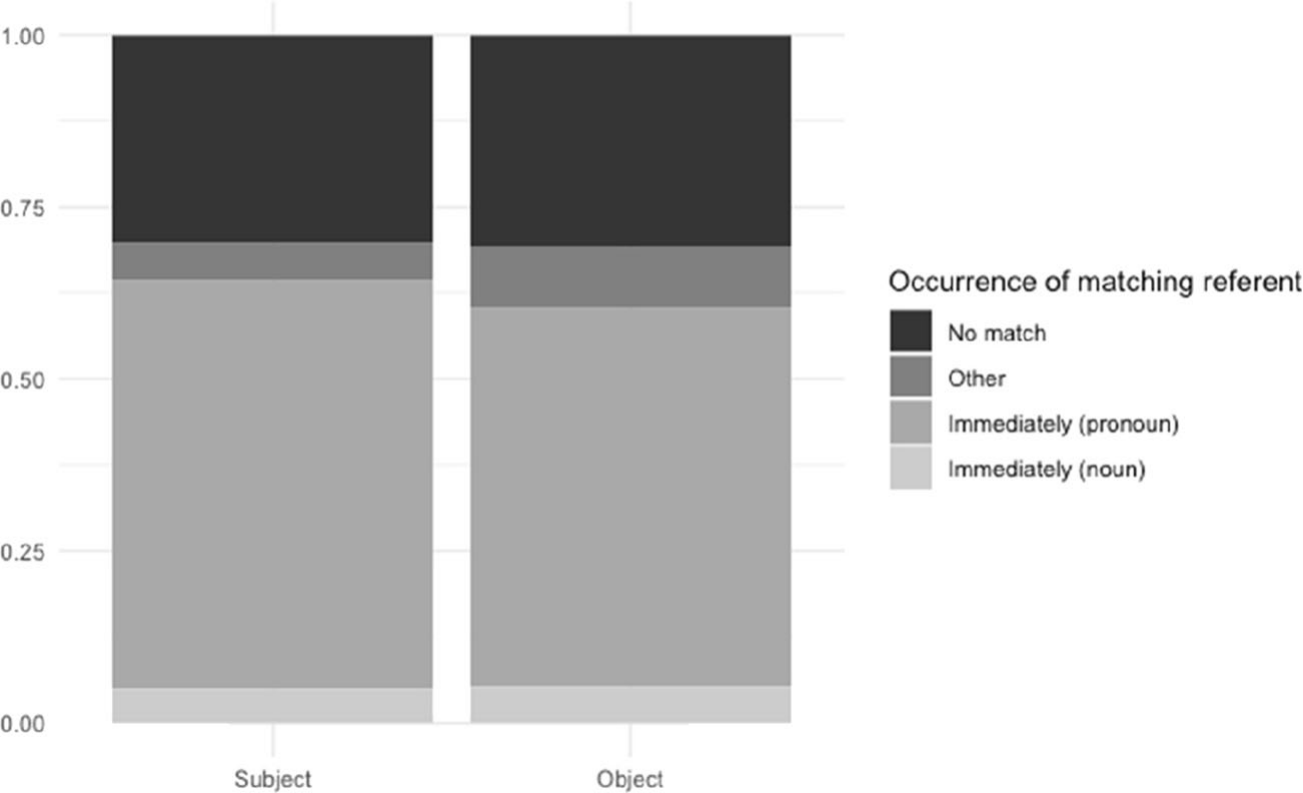
Experiment 1B: Responses for subject and object position

Participants showed a preference for immediate matches: in 62.2% of the responses, the first available argument position contained a co-referent element. This element was a pronoun in 91.8% of the cases. In 7.3% of the responses, a co-referent element occurred not immediately, but in another position; for 30.4%, there was no match in the response (see Fig. 3). Although an immediate match occurred slightly more often in subject position, the difference with object position was minimal (generalized mixed-effects model with position as fixed effect; subject and item as random effects: *z* = 0.62).

#### Discussion

The expectation of coreference between the cataphor and a noun phrase in upcoming positions suggested by the gender mismatch effects in the self-paced reading results is also reflected in the sentence completions. Participants tended to introduce a co-referent element in their sentence completion responses as soon as possible, regardless of position. This preference is in line with an active and persistent parser: we see only a small proportion of responses in which the co-referent element, if present, occurs in another position than the first available argument position.

The large proportion of pronouns instead of lexical nouns in the *immediate match* responses seems surprising from the perspective of the persistent search hypothesis. If the occurrence of a cataphor causes the parser to actively search for an antecedent merely in order to interpret the cataphor and establish its referent, we might expect more responses introducing an antecedent, in the form of a lexical noun. The clear preference instead for pronouns raises the question whether active search responsible for the gender-mismatch effects in Experiment 1A may not (merely) be motivated by a need to discharge the cataphoric dependency, but by a coherence-related drive to establish the role of the cataphor’s referent in the main clause. However, the preference for pronouns may also be a task effect. Participants may have occasionally interpreted the isolated sentences as snapshots from an implicit discourse context containing a referent for the cataphor. In addition, participants may have preferred to avoid elaborating the discourse context given in the preamble with as little extra information as possible. Such a tendency could result in a reluctance to invent the identity of the cataphor given in the preamble, and instead, treat the cataphor as a pronoun with an antecedent in an implicit preceding discourse. The same task effect may also have contributed to the sizeable 30% of responses that did not contain a cataphor at all.

**Fig. 4:**
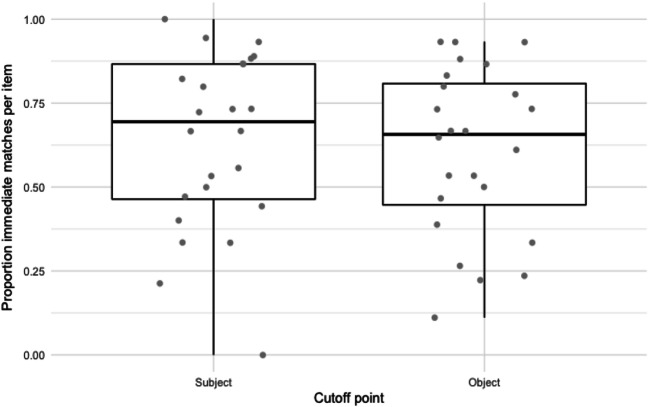
Experiment 1B: Proportion ‘immediate match’ responses by item

Another notable result is that the variability in proportion of *immediate match*-responses between items in subject condition was comparable to the variability in object condition (see Fig. 4). If by-item variability is caused by the factors related to discourse context and plausibility, we should expect more variability in the object condition, because the object preambles provide a richer and more specific context.^5^ An inspection of the individual items showed that among the five items with the lowest proportion of *immediate match* responses in the subject condition (under 0.4) were all three items with the finite verb *begynte*, ‘started to’. All of these three (and only these) had the subordinator *så snart*, ‘as soon as’. Possibly, some property of the subordinator or verb made participants dis-prefer an individual in the subject position. *No match* responses for these three items had either an indefinite plural as the main clause subject (e.g., *as soon as he had left the building, everyone started to laugh*) or an expletive subject (e.g., *as soon as he had left the building, it started to rain*).

### Item-specific resolution probability and gender mismatch effects: Post hoc analysis

In light of the by-item variability in the sentence completion task, we carried out a post hoc analysis on the self-paced reading data to investigate whether item-specific probability of resolving the cataphor modulated the gender-mismatch effect. We took the responses from the sentence completion task and interpreted the proportion of *immediate match* responses for each item (separate for the subject and object cutoff point conditions) as the item-specific probability of immediate match (PIM).

#### Analysis

We analyzed log-transformed self-paced reading times of the critical and spillover regions for subject and object conditions in separate linear mixed-effects models. We fitted linear mixed-effects models with cataphor match, empirical log odds of the subject and object PIM of each item, and their interaction as fixed effects. Models included item and participant as random effects. As before, we used a maximal random effect structure for participant where possible. For Item, we only added random slopes for Match.

#### Results

For the subject conditions, the model for the name region only showed a significant main effect of match (*t* = 2.18, *p* = .039). In the spillover region, this main effect persisted (*t* = 3.49, *p* < .001). We also observed a significant Match × PIM negative interaction (*t* = −2.18, *p* = .03) in the spillover region. The interaction was in an unexpected direction: the gender mismatch effect increased slightly as the PIM decreased. This interaction was largely driven by the effect of four extreme data points with PIM log odds below −5 or above 5.

For the object conditions, the model for the name region did not reveal any significant effects, just like in the main analysis, and a weak numerical trend towards a Match × PIM positive interaction (*t* = 1.40, *p* = .17). As opposed to the main analysis, the spillover region did *not* show a significant main effect of Match (*t* = 1.19, *p* = .24). Instead, the model for the spillover region showed a numerical trend towards a main effect of PIM (*t* = −1.80, *p* = .085) and a Match × RP positive interaction (*t* = 1.97, *p* = .052). Inspection of this interaction revealed that the mismatch effect increased as the PIM increased.

#### Discussion

In the post hoc analysis, we investigated whether the effect of Match observed in the main analysis was modulated by item-specific PIM. Whereas the name region did not reveal an interaction between match and PIM, the model provides weak evidence of interactions in the spillover region in opposite directions for subject and object conditions. The marginally significant interaction for object conditions represents the intuitive correlation that larger gender-mismatch effects are observed in cases where participants were more likely to produce a matching antecedent in an off-line completion task. The interaction in the subject conditions describes a slight decrease in the size of the gender-mismatch effect as the probability of producing a matching antecedent increases. The direction of the interaction in subject conditions is surprising. We reasoned that a stronger expectation of an upcoming co-referent element might lead the parser to commit more strongly to a prediction of such an element, leading to a greater disruption when the prediction is foiled. However, the interaction should be interpreted with caution, since it is mainly driven by a small number of items with extreme PIM values in the subject condition, including the items with the auxiliary *begynte* (‘*started to*’; see Experiment 1B: Sentence Completion Task section). Taken together, the analysis does not provide conclusive evidence that item-specific PIM strongly modulated the observed gender mismatch effects.

## Experiment 2 (English)

The aim of this experiment was to replicate the results from Experiment 1 in another language. In a similar self-paced reading experiment, we investigated whether cataphor gender-mismatch effects past the subject position also occurred in English. The design and analysis of Experiment 2 was preregistered (AsPredicted #38718).

### Experiment 2A: Self-paced reading

#### Materials

Twenty-eight items were constructed with the same 2 × 2 design as in Experiment 1A: singular cataphors in a preposed adjunct clause were manipulated to match or mismatch a proper name, which occurred either in subject or (in)direct object condition (see Table 4).

**Table 4:**
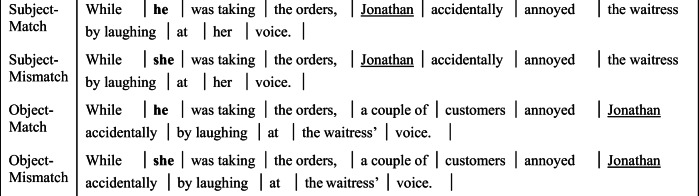
Experiment 2A: Item set

*Note.* Regions separated by vertical bars.Unlike Experiment 1A, the critical name region was always followed by an adverb, making the spillover region identical across all four conditions. This allowed for a direct comparison of the spillover region in subject and object conditions using a single linear mixed-effects model. An additional advantage of the adverb is that the spillover region for the subject conditions was not the main clause verb, as it was in Experiment 1A, avoiding any influence of the processing or integration of the main verb and its argument structure in this region.

As in Experiment 1A, the preposed adjunct clause introduced no explicit additional referents. The items were constructed such that in each condition, there was at least one matching candidate antecedent in the main clause. All gender-matching and number-matching referents in the main clause were at least incrementally^6^ plausible antecedents for the cataphor. In the object conditions, the subject spanned two regions and consisted of a plural DP with a quantifier.

The 28 items were combined with 68 fillers of similar length and complexity. The fillers contained sentences with subordinated clauses, proper names, and noncataphoric anaphors. The trials were distributed over four lists and pseudo-randomized for each participant. Unlike Experiment 1A, we did not add extra subject-match cataphor sentences to the fillers.

#### Procedure

The experiment was hosted online on the Ibex platform (Drummond, 2013). Participants were instructed to read the sentences carefully, at a pace that allowed for a natural interpretation. Before the experiment started, participants completed multiple practice trials. The experiment was divided in blocks of 24 sentences, separated by a ‘break’ screen.

The presentation method differed slightly from Experiment 1A. In Experiment 2A, regions were displayed centered in a noncumulative self-paced reading fashion such that participants did not see previous or upcoming masked regions of any sentence. We opted for centered presentation to ensure that the stimuli looked maximally similar for all online participants with different monitor types.

Trials started with a 1,500-ms display of a fixation cross. Participants moved through the sentence by pressing the space bar. Each sentence was followed by a yes–no comprehension question, which never targeted the interpretation of the cataphor. Incorrect answers were followed by direct feedback and a reminder to read attentively. After completing the experiment, participants filled out a small demographic survey, which we used for screening purposes.

#### Participants

Ninety participants were recruited via Prolific Academic (https://www.prolific.co) and participated online via a link to the experiment. They received 4.00 GBP for participating. The 80 participants that met the inclusion criteria (see below) had a mean age of 32.

#### Predictions

Based on the results from Experiment 1, we predict gender-mismatch effects for both subject and object conditions, in line with the persistent search hypothesis. As in Experiment 1, we expect the effect to take the form of longer reading times at the proper name and/or at the first spillover region, when the proper name mismatches the gender of the cataphor.

#### Analysis

Nine participants who answered correctly on less than 80% of the comprehension questions of the fillers were excluded. In addition, we excluded one participant who reported being a nonnative speaker of English. For the remaining 80 participants, the mean accuracy for the filler items was 92% (range: 81%–100%). Reading times under 100 ms and over 4,000 ms were excluded (<0.1% of the data).

We analyzed log-transformed reading times for the critical name region and the spillover region. Using the lme4 and lmerTest package in R, we ran linear mixed-effects models for both regions, with match (match/mismatch), position (subject/object), and their interaction as fixed effects, and random intercepts for subject and item. Random slopes for match and position were always included by subject and item. Random slopes for the Match × Position interaction were included whenever the models converged.

#### Results

##### Comprehension questions

The mean accuracy for comprehension questions following the fillers was 0.92 and for the experimental items 0.90. There was no significant difference between the accuracy for items and fillers, nor was there significant variation between experimental conditions (generalized mixed-effect models with item and participant as a random effect).

##### Reading times

**Fig. 5:**
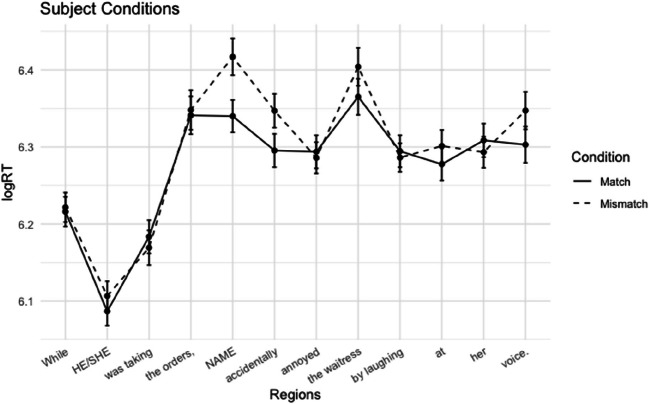
Experiment 2A: Average log reading times + standard errors for subject conditions

**Fig. 6:**
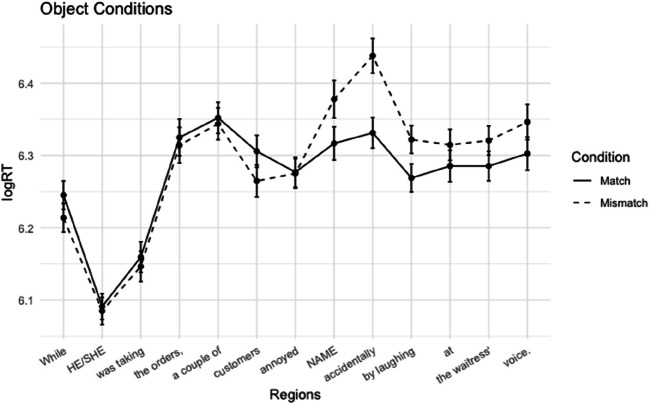
Experiment 2A: Average log reading times + standard errors for object conditions

Average log-transformed reading times for subject and object conditions are plotted in Figs. 5 and 6. Average reading times for the critical regions are displayed in Table 5. In the critical name region, there was a significant main effect of Match, with longer reading times for the mismatch conditions (*t* = 2.52 *p =* .01). There was no significant main effect of Position, nor a Match × Position interaction.

**Table 5:** Experiment 2A: Mean reading times in ms (*SE*) for the critical name regions and the subsequent regions

	Name region	Spillover region
Subject-Match	647 (17)	629 (19)
Subject-Mismatch	728 (21)	656 (16)
Object-Match	654 (20)	646 (17)
Object-Mismatch	733 (26)	751 (24)

In the spillover region, we see a similar slowdown for the mismatch conditions, resulting in a significant main effect of Match (*t* = 4.40, *p* < .001). The model did not yield a significant main effect for Position or a Match × Position interaction, although there was a trend towards an interaction (*t* = −1.91, *p* = .06), driven by a larger mismatch effect in object position. Pairwise comparisons for subject and object conditions show mismatch effects for both positions in the name region (difference of least squares means: respectively *p* = .004 and *p* = .013). For the spillover region, the pairwise comparison also show significant mismatch effects for both the subject conditions (*p* = .04) and object conditions (*p* < .001).

#### Discussion

We observed main effects of Match in both the critical name region and the spillover region. These main effects of Match did not interact with Position in either region, suggesting that similar gender-mismatch effects occurred in subject and object position. As such, we replicated the results from Experiment 1A, which also showed gender-mismatch effects in both subject and object position. The gender-mismatch effect in object position may be taken as evidence against the limited prediction hypothesis: The results indicate that the parser engages in an active antecedent search that updates as the sentence unfolds and persists past the subject, as suggested by the persistent search hypothesis.

We did not replicate the difference in effect size suggested by Experiment 1A. Numerically, the effect in Experiment 2A was even larger in object position than in subject position. This suggests that the apparent difference in effect size between subject and object in Experiment 1B was not caused by the position of the antecedent. Similar to Experiment 1, the effect seems to occur later in object position, reaching significance first in the spillover region. It is unclear to us how strongly to interpret the differences in the onset region of the gender-mismatch effect. One possible explanation for a later effect in object position could be that prediction is less ‘certain’: the parser might commit less strongly to predicted coreference in object position than the prediction in subject position. Prediction strength could be influenced by a number of factors, including verb subcategorization or selectional restrictions, or the fact that a previous prediction was disconfirmed in the same sentence.

Overall, Experiment 2 yielded slightly more robust mismatch effects than Experiment 1. A possible factor contributing to this difference may be the different presentation modes. As a reviewer pointed out, it is possible that the fully masked presentation in Experiment 1 affected participants’ willingness to commit to a prediction of an antecedent in object position if they judged that the visible remainder of the sentence was long enough to plausibly contain another potential antecedent position. The presentation mode may have contributed to the stronger overall effects in Experiment 2, but on a more fine-grained level, it does not explain the different patterns of relative subject-effect and object-effect size between the experiments. If participants strategically modified their (degree of commitment to) a prediction using the amount of upcoming material, we should find larger effects in object position for Experiment 1. The less upcoming material that remains in the sentence, the more reason a reader might have to expect (and commit to) an antecedent in an upcoming position. We observed, however, the reverse image: larger subject effects in Experiment 1, and larger object effects in Experiment 2.

### Experiment 2B: Sentence completions

To investigate whether the gender mismatch effects in the self-paced reading experiment were modulated by item-specific resolution proportions in this experiment, we created a sentence completion experiment based on the English items, along the lines of the Norwegian Experiment 1B.

#### Materials and procedure

The materials were constructed from the self-paced reading items the same way they were constructed for the Norwegian experiment. Forty-nine fillers were added to the four lists, and the experiment was carried out with the same procedure as the Norwegian experiment. Participants were recruited via Prolific Academic and received GBP 3.50 for participation. The experiment took approximately 25 minutes.

#### Participants

Twenty-nine native speakers of English between age 18 and 50 (mean age: 30) were recruited via Prolific Academic. Two participants were excluded because of a large number of ungrammatical and nonsensical responses.

#### Results

**Fig. 7:**
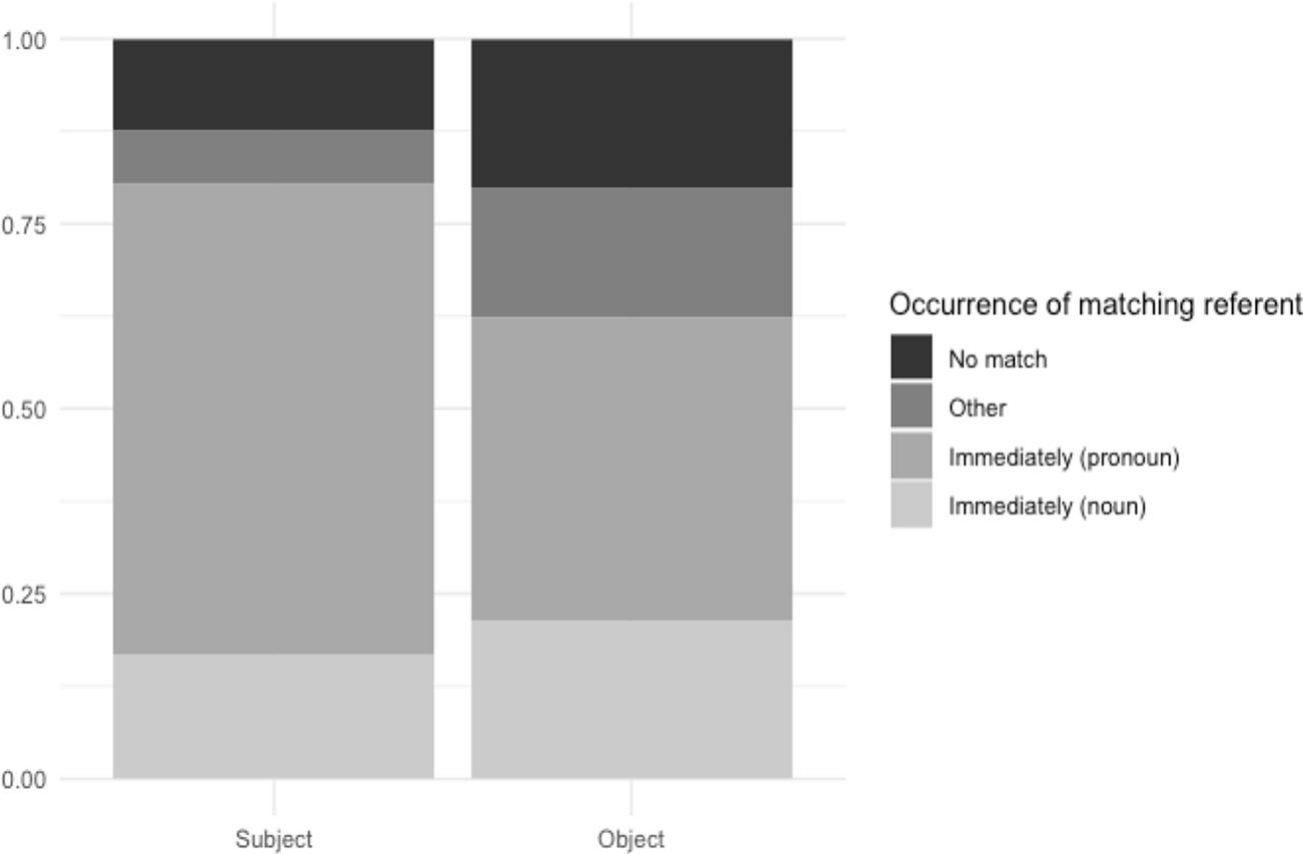
Experiment 2B: Responses for subject and object position

The responses were coded in the same way as for the Norwegian data. The responses per condition and per item are plotted in Fig. 7.

The subject conditions yielded significantly more *immediate match* responses than the object conditions did (generalized mixed-effects model with position as fixed effect, participant and item as random effects; *z* = 3.11, *p* < .01). The object conditions had both more *other match* and more *no match* responses.

**Fig. 8:**
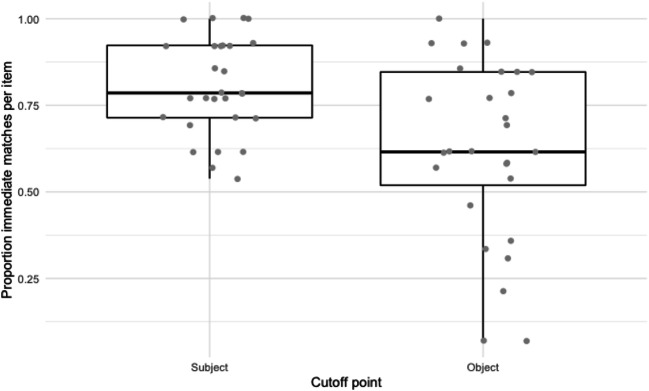
Experiment 2B: Proportion ‘immediate match’ responses by item

In both conditions, the *immediate match* responses consisted of mostly pronouns, but the proportion *immediate match* nouns in this position was considerably higher than we saw in the Norwegian experiment (20.68% vs. 7.54% for subject position; 33.91% vs. 8.86% for object position). As illustrated by Fig. 8, the by-item variability in proportion of *immediate matches* is larger in the object conditions than in the subject conditions.

#### Discussion

The results of the English sentence completion experiment are similar to the Norwegian results in a number of respects. In both experiments, we see a clear preference for introducing a co-referent element in the first available argument position. Also similar to the Norwegian results, pronouns made up the majority of the *immediate matches*.

A difference with the Norwegian data is the by-item variability: whereas the Norwegian results showed similar variability across conditions, the English data show a greater variability for object conditions than for subject conditions. As suggested in Experiment 1B: Sentence Completion Task section, this greater variability is likely a consequence of the richer context given in the preamble for the object conditions: the extra elements (main clause subject and verb) in each item set up a more specific context, with a more specific plausibility of the cataphor and object being co-referent.

### Post hoc analysis on self-paced reading times

We carried out the same analysis as we did for Experiment 1 to test for the influence of item-specific proportion of immediate matches (PIM) on the gender-mismatch effect. Again, we used the empirical log odds of PIM as a predictor. Analysis was identical to that in Experiment 1B.^7^

#### Results

In the name region, the main effect of match was significant (*t* = 2.97, *p* = .004). Neither the effect of PIM nor the Match × PIM interaction was significant (*t* = 0.25 and *t* = 0.01, respectively). In the spillover region, we see again only a significant main effect of match (*t* = 4.8, *p* < .001). There was no significant main effect of PIM (*t* = ×1.12), nor a Match × PIM interaction (*t* = −1.42).

#### Discussion

We observed main effects of Match, indicating that gender-mismatch effects in the self-paced reading experiment occurred regardless of item-specific PIM. The models showed no significant effects of PIM on the reading times, and do not provide evidence that the observed online gender mismatch effects are modulated by item-specific PIM. These results are consistent with an active parser whose (degree of commitment to) predictions is not strongly influenced by the minimal discourse context and plausibility of the current sentence. However, it is also possible that the off-line responses that constituted PIM do not properly capture information available during online processing.

## General discussion

The present study aimed to determine whether predictive antecedent search during the processing of cataphoric pronouns can be characterized as *persistent*. We defined antecedent search as *persistent* if the parser continued to actively posit a cataphor’s antecedent in later positions after its first prediction was disconfirmed. To test for persistence, we had participants read sentences containing a cataphor in a fronted adjunct clause. We used a gender-mismatch manipulation to investigate in which syntactic positions we could find signs of active antecedent search. In two self-paced reading studies, we observed gender mismatch effects at the main subject immediately following the fronted adjunct. These results are consistent with previous cross-linguistic work indicating that readers actively search for an antecedent in the main subject position (Drummer & Felser, 2018; Kazanina et al., 2007; Kazanina & Phillips, 2010; Pablos et al., 2015; van Gompel & Liversedge, 2003). Important for our purposes, we also found gender-mismatch effects in the postverbal object position in the same sentences when the main subject did not provide a suitable antecedent for the cataphor. These results indicate that gender mismatch effects in previous studies cannot be explained as arising from a single prediction of coreference with an upcoming subject position. Instead, they suggest that the incremental parser continuously updates its predictions for potential antecedents in upcoming syntactic positions, in line with the persistent search hypothesis.

Kazanina et al. (2007) proposed that antecedent search in cataphoric processing could be analogized to more familiar active dependency creation operations, like active gap-filling in filler-gap dependency processing. Such a proposal suggests that both procedures may employ the same underlying general-purpose active search mechanism. We reasoned that the analogy was apt insofar as both search mechanisms exhibit the same behavioral characteristics. Adding persistence to the similarities between cataphor antecedent search and active gap-filling provides support for the shared mechanism hypothesis.

### Implications for models of active dependency completion

In the past, there have been debates about what principles drive active gap-filling, or active dependency completion more generally (Aoshima et al., 2004; Keshev & Meltzer-Asscher, 2020; Stowe, 1986; Wagers & Phillips, 2009). One camp ties active dependency completion to *syntactic* pressures—comprehenders attempt to discharge syntactic dependencies as quickly as possible. Under such an account, active search is employed only when the second part of a dependency is obligatory for the syntactic well-formedness of the sentence. Another possibility is that active dependency completion stems from a general preference for discharging *all* grammatical dependencies as early as possible and with a minimum of structural commitments (‘minimal everything’; Fodor, 1998; Inoue & Fodor, 1995).

Our results suggest that active dependency completion can be driven by a general grammatical pressure to resolve dependencies as quickly as possible. Active gap-filling may be driven by a syntactic requirement to find a gap for an unresolved filler. Active antecedent search, on the other hand, might be thought to arise from the *pragmatic* requirement that all pronouns have an antecedent in the local discourse context: the use of pronouns without a (topical) antecedents is pragmatically infelicitous (Grosz et al., 1995; Hankamer & Sag, 1976).^8^ Insofar as our sentences are presented in isolation, the only discourse context comprehenders have access to comes later in the sentence.

Assuming that a single active and predictive mechanism underlies cataphor processing and filler-gap processing alike has implications for how the mechanism should be characterized. If active dependency completion is also observed with cataphora, active search cannot be exclusively dependent on the syntactic obligatoriness of the second element. Instead, it suggests that the pressure to discharge syntactic dependencies as quickly as possible is part of a more general pressure to maximize incremental well-formedness (Aoshima et al., 2004; Wagers & Phillips, 2014).

The idea that pragmatic or nonsyntactic pressures can trigger active dependency completion comparable to tendencies observed in gap-filling might first appear at odds with recent results from Keshev and Meltzer-Asscher (2020). The authors found that topicalizing a referent inside a sentence did not create strong expectations for a co-referent NP in a later position in the same way that a *wh*-filler created an expectation for a gap. The authors observed strong evidence for rapid active-gap filling (filled gap effects), but effects of referential resolution were smaller and occurred later. Specifically, they observed longer reading times for NPs introducing a new referent in the object position when they were preceded by a topicalized referent. The pragmatic expectation that a sentence is ‘about’ a topicalized referent thus yielded smaller and later effects than filled *wh*-gaps, which might suggest that pragmatically conditioned dependency completion is somehow “less active” than active gap-filling.

We suggest that active search—or the degree of activity—may vary as a function of the strength of the grammatical well-formedness condition that motivates dependency formation or completion. The syntactic requirement of gaps in filler-gap dependencies and the pragmatic requirement for pronouns to have a topical antecedent are both relatively strong conditions for broad well-formedness, triggering a high degree of active search. On the other hand, in Keshev and Meltzer-Asscher’s pragmatic manipulation items, there was arguably not a strong pragmatic requirement that the topicalized referent be mentioned again later in the sentence. We suggest that the smaller effects they observed for their pragmatic manipulation may reflect weaker active search, triggered by a weaker well-formedness pressure.

How exactly to cache out the notion of varying degrees of activity remains to be determined. There are different ways of implementing variable prediction strength that can lead to variable effect sizes. It is possible that strength of prediction influences the commitment of the parser to the prediction, and the amount of resources it consequently dedicates to the active search (see Kuperberg & Jaeger, 2016 for a review). Another similar idea is that strength of prediction at a high level reflects the number of smaller representational commitments the parser makes that are entailed by the larger umbrella prediction. Recovery from a false prediction would lead to a larger disruption for a highly committed parser, and to a greater, and perhaps more immediate, effect size. Another parsing mechanism that would lead to variable effect sizes is a probabilistic mechanism that decides to engage in active search only some of the time, informed by probabilities of a successful prediction. Such probabilities may be informed by the frequency of grammatical structures (e.g., a subject gap, or subject coreference; see Wagers & Pendleton, 2016, for a proposal of this type). If the probability of active search depends on the probability of successful prediction, lower probabilities of success would lead to smaller proportions of times that the parser engages in active search, leading to smaller average effect sizes.

#### Antecedent search versus coreference expectation

In line with the previous literature, we have described active search triggered by cataphora as active search for the antecedent. Our findings are also consistent with active search not primarily for an *antecedent* in the form of a lexical noun or name, but for any co-referent element generally, which also includes another pronoun. Such a prediction may be driven by a desire to quickly establish coherence between the subordinated and main clause and incrementally interpret the proposition conveyed by the sentence. The preference for pronouns rather than lexical nouns in the sentence completion responses are consistent with this suggestion.

However, as discussed, the proportion of pronouns may be explained as a task effect. It may be caused by participants’ reluctance to extend the given discourse context by inventing the antecedent of the cataphors, or by participants’ interpretation of the experimental sentences as being snippets taken out of a discourse context containing appropriate antecedents for the pronouns in the preambles.

## Conclusion

We investigated whether active antecedent search triggered by a cataphor can be characterized as a persistent mechanism that posits co-reference with upcoming syntactic positions after a first prediction of co-reference is disconfirmed. We observed mismatch effects consistent with active antecedent search in main clause object and subject position in the same items within the same study, providing evidence that cataphors trigger a persistent active search mechanism similar to what is assumed about active gap-filling. These similarities suggest that active dependency formation should be viewed as a parsing strategy that (i) is not construction-specific and (ii) does not depend on the syntactic obligatoriness of the dependency.

## Footnotes

1 The two subject control verbs we used were *begynte* (‘started to’) and *prøvde* (‘tried to’).

2 For example, in a sentence (Norwegian word order) like ‘after she had stolen the sunglasses, forbade …’, comprehenders are, in principle, able to adjust their expectance of coreference with the main clause subject based on the plausibility of the embedded subject being the subject/Agent of *forbade*.

3 A property of the design is that in three of the four conditions, the main clause subject mismatched the gender of the cataphor. As an additional measure to counteract participants developing an expectation of unresolved pronouns, we compensated for the imbalance between matching and mismatching main clause subjects by adding 10 subject-match cataphor sentences in the fillers. Reading times for these extra cataphor sentences were not analyzed.

4 We interpreted ‘intended co-reference’ liberally: Morphologically matching referents were only classified as ‘not co-referent’ in overwhelmingly clear cases where plausibility dictated noncoreference (21 responses). An example: *after he paid, the cashier gave Eric the receipt.* In this response, *the cashier* was classified as ‘not co-referent’. Although cataphoric reference is inherently ambiguous, in most responses, there was little doubt about the intended coreference. Typical responses only contained one referent, which was not only a morphological match for the cataphor, but also a plausible antecedent.

5 This is the pattern we find in the English sentence completion study (Experiment 2B; see Experiment 2B: Sentence Completions section).

6 In 5 of the items, a referent past the critical proper name might ultimately be the more plausible referent (e.g., *After he turned up the volume, David energetically signaled to the DJ…*). However, this could not affect real-time reading times on the critical regions preceding this referent.

7 Although we planned to carry out this analysis with *z*-scaled PIM scores (see preregistration), we chose to use empirical log-odds instead as a more standard and more suitable measure for this analysis. The results we report here are qualitatively similar to the analysis with *z*-scaled PIM scores.

8 Except for nonanaphoric uses of pronouns, such as the impersonal use of *they*.
